# Use of Low Molecular Weight Heparin and Aminocaproic Acid in Chronic DIC Associated with Prostate Cancer- A Case Report

**DOI:** 10.1100/tsw.2007.143

**Published:** 2007-02-02

**Authors:** Keisuke Shirai, Uzair B. Chaudhary

**Affiliations:** Department of Medicine, Division of Hematology/Oncology, Medical University of South Carolina, Charleston, SC, USA

**Keywords:** DIC, prostate cancer, aminocaproic acid, low molecular weight heparin

## Abstract

Disseminated Intravascular Coagulopathy (DIC) is the most common coagulopathy in patients with prostate cancer. Though rare, it could be fatal without treatment. Literature suggests that there is significant activation of fibrinolytic pathway. Pathophysiology of DIC in patients with prostate cancer is not completely understood. We present here a case of chronic DIC in a patient with metastatic androgen independent prostate cancer. His DIC was managed successfully with a combination of aminocaproic acid and low weight molecular heparin. The use of low molecular weight heparin may make management of chronic DIC in prostate cancer more feasible in an out patient setting.

## INTRODUCTION

DIC is a life threatening coagulopathy seen in prostate cancer. The pathophysiology is not yet fully understood. The main laboratory findings are thrombocytopenia, prolonged prothrombin time (PT), prolonged partial thromboplastin time (PTT), low fibrinogen and elevated fibrin degradation products (FDP). The mainstay of treatment of DIC is to control underlying disease. Docetaxel based chemotherapy is often used in advanced metastatic androgen independent prostate cancer (AIPC). However, control of DIC is usually problematic. We report a case of DIC in a patient with metastatic AIPC that was successfully treated with aminocaproic acid in combination with subcutaneous low molecular weight heparin injection in an outpatient setting.

## CASE REPORT

A fifty four-year old African American male with metastatic AIPC was admitted to our hospital with history of 4-5 days of epistaxis and fatigue. Physical examination was unremarkable. Laboratory work-up revealed a white blood cell count of 10x $10^3/mm^3$, hemoglobin of 8.7 gm/dl, platelet of $31x10^3/mm^3$, fibrinogen was less than 60 mg/dl, International Normalized Ratio (INR) 2.2, PT 24 seconds, PTT 33 seconds and serum creatinine 0.8 mg/dl.

He was initially diagnosed with prostate cancer in 1997 with a Gleason score of 8 and a serum prostate specific antigen (PSA) of greater than 50 ng/ml. He was treated with leuprolide and external beam radiation therapy. Initially, his PSA became undetectable, but in 2000 it began to rise. Combined androgen blockade with leuprolide and bicalutamide was reinitiated with minimal response. In 2001, despite use of ketoconazole treatment, his PSA continued to rise and he was found to have bone metastase in the thoracic and lumber spine.

## Hospital course

He was supported with packed red blood cells, platelets, and cryoprecipitate. He was started on weekly docetaxel (35mg/m$^2$), on a day 1, 8 schedule every three weeks. He required daily platelet transfusions and cryoprecipitate to maintain his platelet count above 30x10$^3$/mm$^3$ and fibrinogen of more than 100 mg/dl. With the initiation of docetaxel treatment, epistaxis resolved with an improvement in his coagulation parameters. After 72 hours he was discharged to home.

Unfortunately 1 week later, he was readmitted to the hospital with epistaxis. At this time fibrinogen was again less than 100 mg/dl with prolonged PT/PTT. To control bleeding, he was treated with 2 g every 6 hours of aminocaproic acid along with subcutaneous low molecular weight heparin (LMWH) 30 mg twice a day. With this combination his platelet and fibrinogen were stabilized in two days (Figure 1). At the time of discharge the dose of aminocaproic acid was tapered to 1 g every 6 hours. The patients DIC parameters were under control for three months with aminocaproic acid at 500 mg every 6 hours and LMWH at 30 mg twice a day without any evidence of bleeding or thrombosis.

## DISCUSSION

Although pathogenic mechanisms of DIC in prostate cancer are not well understood, an overly activated fibrinolytic pathway is thought to be the cardinal feature of this condition[1,2]. Fibrinolytic bleeding could also be seen in other neoplastic diseases such as carcinoma of the lung, stomach, and cervix. With an activated fibrinolytic pathway patients can have fatal bleeding. Early recognition of DIC is essential to avoid a fatal course.

Several treatment approaches to control DIC in prostate cancer have been described in the past. Needless to say, the most important treatment is to control underlying disease. Chemotherapy with docetaxel or mitoxantrone plays a key role in metastatic AIPC[3,4]. A variety of hormonal maneuvers including DES[5,6] and ketoconazole[7,8] have been used with limited success. Several successful case reports with radioactive agents including samarium 153 have been reported with limited success[9].

The patient reported here responded initially to treatment with docetaxel only for a short period of time. However, he was readmitted with bleeding and abnormal coagulation parameters. He required multiple cryoprecipitate and platelet infusions to control bleeding and maintain fibrinogen level above 100 mg/dl.

In 1992, Cooper et al. reported a successfully treated DIC case in prostate carcinoma with aminocaproic acid and low dose heparin continuous infusion (300-500units/hour)[10]. Aminocaproic acid was chosen to control an overactivated fibrinolytic pathway and at the same time low dose heparin was given to compensate for the hypercoagulable state induced by aminocaproic acid. As far as we know, this is the only case report of DIC in prostate cancer effectively treated with aminocaproic acid.

Aminocaproic acid is 6-aminohexanoic acid, which acts as an inhibitor of fibrinolysis. Aminocaproic acid is useful in enhancing hemostasis when fibrinolysis contributes to bleeding. It is indicated in bleeding patients with hemophilia. Low molecular weight heparin subcutaneous injection is now available and can replace continuous low dose heparin infusion. With the combination of oral aminocaproic acid and subcutaneous low molecular weight heparin, chronic DIC in prostate cancer can be successfully managed in an outpatient setting feasibly as described above.
